# Longitudinal trends in antimicrobial resistance phenotypes in *Pseudomonas aeruginosa*: a seven-year study

**DOI:** 10.3389/frabi.2026.1912287

**Published:** 2026-09-18

**Authors:** Wael A. Alghamdi, Khalid M. Orayj, Ahmed R. N. Ibrahim, Marim A. Abdelsalam, Abdullah M. Alshehri

**Affiliations:** 1 Department of Clinical Pharmacy, College of Pharmacy, King Khalid University, Abha, Saudi Arabia; 2 Faculty of Pharmacy, Minia University, Minia, Egypt; 3 Department of Microbiology, Khamis Mushait General Hospital, Khamis Mushait, Saudi Arabia

**Keywords:** antimicrobial resistance, difficult-to-treat resistance, extensively drug-resistant, multidrug resistance, *Pseudomonas aeruginosa*

## Abstract

**Background:**

*Pseudomonas aeruginosa* is a major cause of healthcare-associated infections and is increasingly associated with antimicrobial resistance. Continuous local surveillance is essential to monitor evolving resistance patterns and inform empirical antimicrobial therapy and antimicrobial stewardship.

**Methods:**

This retrospective laboratory-based surveillance study analyzed non-duplicate *P. aeruginosa* isolates recovered from hospitalized patients at a secondary healthcare setting in Saudi Arabia between 2018 and 2024. Resistance phenotypes were classified as multidrug-resistant (MDR), extensively drug-resistant (XDR), and difficult-to-treat resistance (DTR) according to established international definitions. Temporal trends were evaluated using logistic regression.

**Results:**

A total of 368 non-duplicate *P. aeruginosa* isolates were included. Respiratory tract specimens were the most common source of isolates. MDR prevalence increased from 51.0% in 2018 to a peak of 87.3% in 2022 before declining to 69.6% in 2024, while XDR reached its highest prevalence of 68.3% in 2023 before declining to 39.1% in 2024. When year was analyzed categorically, significant differences across years were observed for MDR (P < 0.001) and XDR (P = 0.003), but not DTR (P = 0.144).

**Conclusion:**

This seven-year surveillance study demonstrated a substantial burden of antimicrobial resistance among *P. aeruginosa* isolates, with significant year-to-year variation in MDR and XDR prevalence. These findings provide locally relevant surveillance data that may inform empirical antimicrobial therapy, antimicrobial stewardship, and ongoing institutional resistance surveillance.

## 1 Introduction

Antimicrobial resistance is a major global public health threat that compromises the effectiveness of antimicrobial therapy and increases morbidity, mortality, and healthcare costs worldwide (Antimicrobial Resistance Collaborators, 2022). Among Gram-negative bacterial pathogens, *Pseudomonas aeruginosa* is a major cause of healthcare-associated infections, including ventilator-associated pneumonia, bloodstream infections, catheter-associated urinary tract infections, and surgical site infections, particularly among critically ill or immunocompromised patients (Moradali et al., 2017). Due to its clinical impact and high resistance potential, *P. aeruginosa* has been classified by the World Health Organization among the critical priority pathogens for the development of new antimicrobial agents (Mancuso et al., 2021).

Treatment of *P. aeruginosa* infections is particularly challenging because this organism possesses multiple intrinsic and acquired resistance mechanisms, including low outer-membrane permeability, multidrug efflux pumps, target-site alterations, and production of β-lactamases. These mechanisms facilitate the emergence and dissemination of multidrug-resistant (MDR), extensively drug-resistant (XDR), and difficult-to-treat resistance (DTR) phenotypes, which markedly limit therapeutic options and are associated with prolonged hospitalization, increased healthcare costs, and higher mortality (Kadri et al., 2018).

In Saudi Arabia and the broader Arabian Gulf region, increasing antimicrobial resistance among *P. aeruginosa* isolates has been documented in recent years. A recent nationwide multicenter surveillance study reported high levels of resistance among clinical isolates, with 25.8% classified as DTR and 4.5% as pandrug-resistant in the southern region of Saudi Arabia (Thabit et al., 2024). Regional analyses across Gulf countries have likewise reported increasing resistance to several antipseudomonal agents, including carbapenems, fluoroquinolones, and aztreonam (Alatoom et al., 2024). These findings highlight substantial heterogeneity in resistance epidemiology across healthcare institutions, emphasizing the importance of local surveillance to guide empirical antimicrobial therapy and stewardship interventions.

Saudi Arabia has implemented national initiatives to address antimicrobial resistance, including the National Action Plan for Antimicrobial Resistance (2022–2025), which aims to strengthen surveillance systems, antimicrobial stewardship, and infection prevention programs nationwide (Saudi Public Health Authority, 2022). Despite these efforts, localized epidemiological data remain limited in some regions, particularly from secondary care settings that are less frequently represented in national surveillance reports.

Continuous institutional surveillance is therefore essential to characterize local resistance epidemiology, monitor temporal trends, and inform empirical antimicrobial therapy and antimicrobial stewardship interventions. Accordingly, this study evaluated temporal trends in *P. aeruginosa* isolates recovered over a seven-year period from a secondary healthcare setting in Saudi Arabia, with particular emphasis on MDR, XDR, and DTR phenotypes. Unlike national and regional surveillance studies that aggregate data across multiple institutions or geographic areas, this study provides longitudinal, institution-level data from a secondary healthcare setting, using patient-level deduplication and standardized resistance phenotype definitions across seven consecutive years. The inclusion of DTR alongside MDR and XDR further provides a clinically relevant assessment of changes in resistance phenotypes over time.

## 2 Methods

### 2.1 Study design and data collection

This retrospective study was conducted at a secondary care hospital in the Aseer region of Saudi Arabia. Clinical microbiology records from 1 January 2018 to 31 December 2024 were reviewed to identify hospitalized patients with cultures yielding *P. aeruginosa*. Ethical approval for this study was obtained from the Aseer Institutional Review Board (approval number: F10-2-2025). Patient confidentiality was maintained by anonymizing all data prior to analysis.

All clinical *P. aeruginosa* isolates recovered from hospitalized patients during the study period were eligible for inclusion. Demographic and clinical data were obtained from laboratory and hospital records and included age, sex, nationality, and specimen source. Specimen sources were categorized into four groups: blood and sterile body fluids, respiratory tract, urine and genital tract, and wound, pus, and tissue.

To minimize duplicate sampling bias, isolates were deduplicated at the patient level. For each calendar year, only the first *P. aeruginosa* isolate per patient, determined by specimen collection date, was included. When multiple specimens were collected from the same patient on the same date, the isolate from the most clinically significant source was retained, with priority given to blood and sterile body fluids over respiratory, urine, and superficial wound specimens. Subsequent isolates from the same patient within the same calendar year were excluded regardless of changes in antimicrobial susceptibility profiles, ensuring that each patient contributed only one isolate per year to the analysis. Because the same patient could contribute one isolate in different calendar years, a sensitivity analysis was also performed using only the first isolate per patient across the entire seven-year study period.

### 2.2 Bacterial identification and antimicrobial susceptibility testing

Bacterial identification and antimicrobial susceptibility testing were performed using the MicroScan^®^ automated broth microdilution system throughout the study period. Antimicrobial panels and routine laboratory procedures followed the clinical microbiology laboratory’s standard practices in effect at the time of testing. Susceptibility results were interpreted according to the breakpoints established by the Clinical and Laboratory Standards Institute (CLSI) and implemented by the clinical laboratory at the time of testing (Clinical and Laboratory Standards Institute, 2025). For analysis, isolates categorized as intermediate or resistant were grouped as non-susceptible.

### 2.3 Definitions of resistance phenotypes

MDR isolates were defined as non-susceptible to at least one agent in three or more antimicrobial categories, while XDR isolates were defined as non-susceptible to at least one agent in all but two or fewer antimicrobial categories (i.e., susceptible to only one or two categories) (Kadri et al., 2018; Magiorakos et al., 2012). The antimicrobial categories evaluated included antipseudomonal penicillins/β-lactamase inhibitors (piperacillin/tazobactam), antipseudomonal cephalosporins (cefepime and ceftazidime), monobactams (aztreonam), carbapenems (imipenem and meropenem), fluoroquinolones (ciprofloxacin and levofloxacin), and aminoglycosides (amikacin, gentamicin, and tobramycin). XDR isolates were considered a subset of MDR isolates.

DTR was defined as non-susceptibility to the tested antipseudomonal β-lactams (piperacillin/tazobactam, ceftazidime, cefepime, aztreonam, imipenem, and meropenem) and fluoroquinolones (ciprofloxacin and levofloxacin) and was analyzed as a clinically defined resistance phenotype. Overall, 92.5% of potential antimicrobial susceptibility results were available. Missing results were treated as unavailable and were not imputed; phenotype classifications were based on the susceptibility results available for each isolate. Annual prevalence of MDR, XDR, and DTR was calculated using deduplicated patient-year isolates as the denominator.

### 2.4 Statistical analysis

Descriptive statistics were used to summarize demographic characteristics, specimen sources, antimicrobial non-susceptibility, and resistance phenotypes. Categorical variables were presented as frequencies and percentages. Temporal differences in MDR, XDR, and DTR prevalence were initially evaluated with calendar year modeled as a categorical variable to account for the non-linear patterns observed across the study period. To address potential non-independence arising from patients contributing isolates in more than one calendar year, a sensitivity analysis was performed after restricting the dataset to the first observation per patient across the entire study period. This resulted in 352 unique patients with no repeated observations. The multivariable logistic regression analyses were performed using this restricted dataset. Separate multivariable logistic regression models were fitted for MDR, XDR, and DTR, including collection year, age group, sex, nationality, and specimen source as independent variables to account for potential differences in case mix. Overall associations were assessed using likelihood-ratio tests, and adjusted odds ratios (aORs) with 95% confidence intervals (CIs) were reported for individual category comparisons. Annual prevalence estimates are presented with 95% confidence intervals calculated using the Wilson method. Statistical analyses were performed using JMP Pro version 18.0 (SAS Institute Inc., Cary, NC, USA), and a two-sided P value <0.05 was considered statistically significant.

## 3 Results

### 3.1 Patient characteristics and specimen sources

A total of 368 non-duplicate *P. aeruginosa* isolates were included between 2018 and 2024 (**Table 1**). Overall, isolates were recovered more frequently from male patients (68.5%), and nearly half originated from patients aged >60 years (49.7%). Saudi nationals accounted for 78.3% of cases. Respiratory tract specimens were the most common source of isolates (35.1%), followed by urine and genital tract (28.3%) and wound, pus, and tissue specimens (24.7%). Blood and sterile body fluid isolates represented 12.0% of all isolates.

**Table 1:** Demographic characteristics and specimen sources of patients with *P. aeruginosa* isolates.

Characteristic	Overall, n (%) (N = 368)
Sex	
Male	252 (68.5)
Female	116 (31.5)
Age group (years)	
0–18	13 (3.5)
19–30	51 (13.9)
31–45	67 (18.2)
46–60	54 (14.7)
>60	183 (49.7)
Nationality	
Saudi	288 (78.3)
Non-Saudi	80 (21.7)
Source of isolates	
Blood/sterile body fluids	44 (12.0)
Respiratory tract	129 (35.1)
Urine and genital tract	104 (28.3)
Wound, pus, and tissue	91 (24.7)

### 3.2 Temporal trends in resistance phenotypes

The prevalence of MDR, XDR, and DTR phenotypes varied over the study period (**Table 2**; **Figure 1**). MDR prevalence increased from 51.0% in 2018 to a peak of 87.3% in 2022 before declining to 69.6% in 2024. XDR prevalence increased from 33.3% in 2018 to 68.3% in 2023 before declining to 39.1% in 2024. DTR prevalence remained consistently lower than MDR and XDR throughout the study period, ranging from 11.8% in 2018 to a peak of 31.7% in 2023 before declining to 15.2% in 2024.

**Table 2:** Temporal trends in MDR, XDR, and DTR *P. aeruginosa* isolates, 2018–2024.

Resistance phenotype	2018 (n=51)	2019 (n=48)	2020 (n=52)	2021 (n=75)	2022 (n=55)	2023 (n=41)	2024 (n=46)
MDR	26 (51.0) [37.7–64.1]	20 (41.7) [28.8–55.7]	32 (61.5) [48.0–73.5]	50 (66.7) [55.4–76.3]	48 (87.3) [76.0–93.7]	32 (78.0) [63.3–88.0]	32 (69.6) [55.2–80.9]
XDR	17 (33.3) [22.0–47.0]	13 (27.1) [16.6–41.0]	22 (42.3) [29.9–55.8]	36 (48.0) [37.1–59.1]	28 (50.9) [38.1–63.6]	28 (68.3) [53.0–80.4]	18 (39.1) [26.4–53.5]
DTR	6 (11.8) [5.5–23.4]	6 (12.5) [5.9–24.7]	11 (21.2) [12.2–34.0]	19 (25.3) [16.9–36.2]	12 (21.8) [12.9–34.4]	13 (31.7) [19.6–47.0]	7 (15.2) [7.6–28.2]

Data are presented as n (%) [95% CI]. XDR isolates represent a subset of MDR isolates, and DTR isolates represent a clinically defined resistance phenotype.

**Figure 1:**
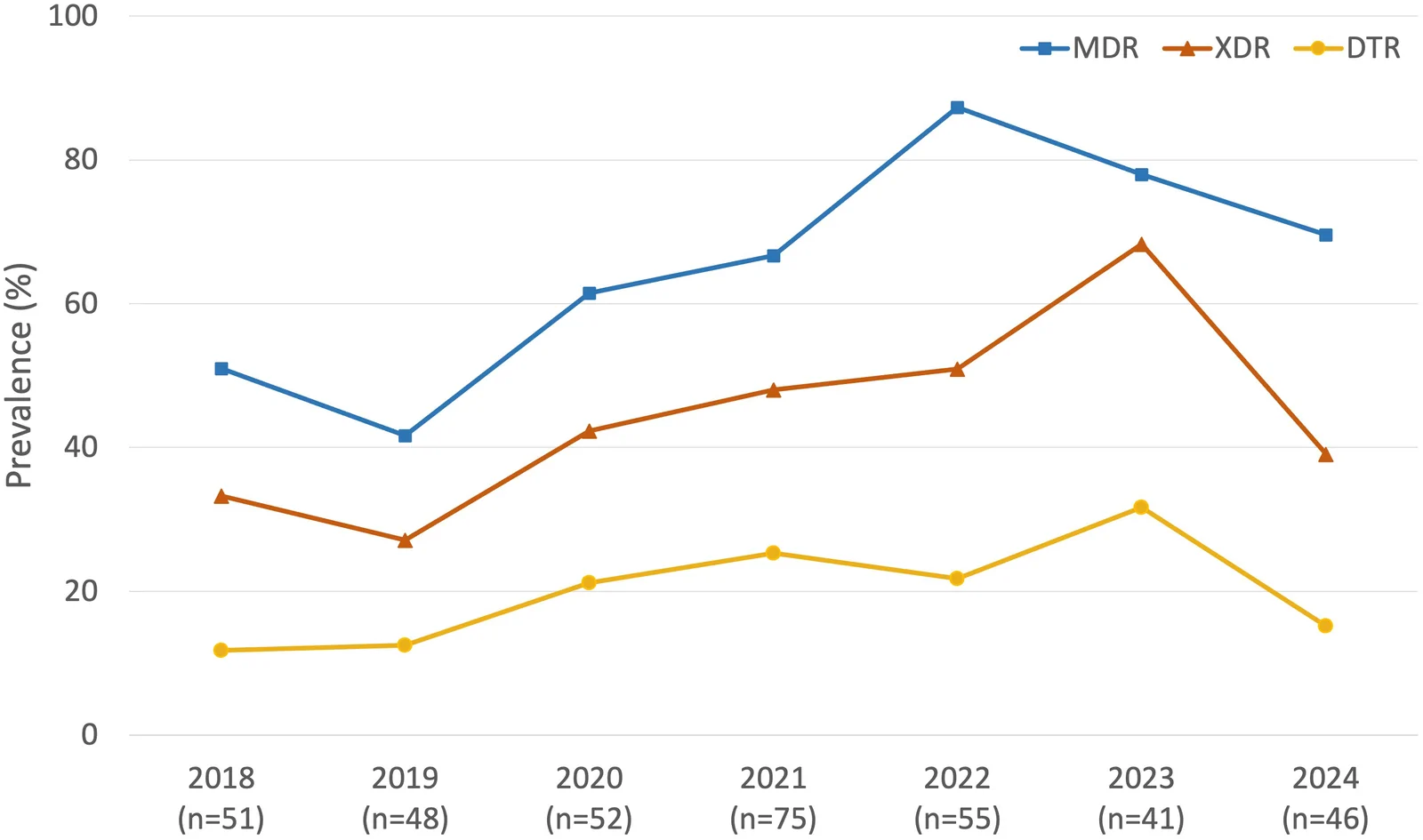
Temporal trends in MDR, XDR, and DTR *P. aeruginosa* isolates, 2018–2024.

When calendar year was analyzed as a categorical variable, significant differences across years were observed for MDR (likelihood-ratio χ² = 33.25, P < 0.001) and XDR (χ² = 20.16, P = 0.003), whereas differences in DTR prevalence were not statistically significant (χ² = 9.57, P = 0.144). In the sensitivity analysis restricted to 352 unique patients, multivariable models adjusting for age group, sex, nationality, and specimen source (**Table 3**) showed that collection year remained significantly associated with MDR (P < 0.001) and XDR (P = 0.013), but not DTR (P = 0.398). Compared with 2018, the adjusted odds of MDR were significantly higher in 2022 (aOR 6.12, 95% CI 2.25–16.63) and 2023 (aOR 2.98, 95% CI 1.13–7.84), while the adjusted odds of XDR were significantly higher in 2023 (aOR 3.59, 95% CI 1.41–9.11). Male sex was associated with higher odds of all three resistance phenotypes, while specimen source was significantly associated with DTR. Antimicrobial-specific resistance also varied over time; notably, ciprofloxacin non-susceptibility increased from 44.0% in 2018 to 97.8% in 2024, while imipenem and meropenem non-susceptibility declined from 45.7% and 41.2% to 30.4% and 27.3%, respectively (**Supplementary Table S1**).

**Table 3:** Adjusted associations of study characteristics with resistance phenotypes.

Variable	MDR aOR (95% CI)	XDR aOR (95% CI)	DTR aOR (95% CI)
Collection year	Overall P <0.001	Overall P = 0.013	Overall P = 0.398
2018	Reference	Reference	Reference
2019	0.60 (0.26–1.39)	0.66 (0.27–1.64)	0.89 (0.25–3.12)
2020	1.47 (0.63–3.42)	1.33 (0.56–3.15)	1.60 (0.50–5.09)
2021	1.99 (0.90–4.37)	1.61 (0.73–3.54)	2.05 (0.71–5.90)
2022	6.12 (2.25–16.63)	1.88 (0.81–4.33)	1.86 (0.60–5.70)
2023	2.98 (1.13–7.84)	3.59 (1.41–9.11)	2.55 (0.80–8.13)
2024	2.04 (0.83–5.03)	1.11 (0.45–2.75)	1.08 (0.30–3.87)
Age group (years)	Overall P = 0.550	Overall P = 0.830	Overall P = 0.805
>60	Reference	Reference	Reference
0–18	0.56 (0.15–2.11)	0.44 (0.09–2.17)	0.51 (0.09–2.87)
19–30	0.56 (0.27–1.17)	1.11 (0.55–2.23)	1.41 (0.59–3.36)
31–45	0.92 (0.45–1.89)	0.96 (0.50–1.87)	1.27 (0.56–2.88)
46–60	0.80 (0.39–1.65)	0.98 (0.49–1.95)	1.05 (0.45–2.44)
Sex	Overall P = 0.047	Overall P = 0.012	Overall P = 0.030
Female	Reference	Reference	Reference
Male	1.72 (1.01–2.93)	1.94 (1.15–3.28)	2.15 (1.05–4.40)
Nationality	Overall P = 0.496	Overall P = 0.789	Overall P = 0.817
Non-Saudi	Reference	Reference	Reference
Saudi	1.25 (0.66–2.35)	1.08 (0.60–1.96)	1.09 (0.53–2.21)
Specimen source	Overall P = 0.330	Overall P = 0.086	Overall P = 0.004
Blood/sterile body fluids	Reference	Reference	Reference
Respiratory tract	1.49 (0.67–3.30)	1.16 (0.55–2.45)	0.49 (0.21–1.16)
Urine and genital tract	1.08 (0.48–2.41)	0.74 (0.34–1.60)	0.25 (0.10–0.63)
Wound, pus, and tissue	0.84 (0.37–1.91)	0.55 (0.25–1.24)	0.24 (0.09–0.61)

aOR, adjusted odds ratio; CI, confidence interval; MDR, multidrug-resistant; XDR, extensively drug-resistant; DTR, difficult-to-treat resistance. Adjusted odds ratios were obtained from separate multivariable logistic regression models for MDR, XDR, and DTR. Each model included collection year, age group, sex, nationality, and specimen source. Overall P values were obtained using likelihood-ratio tests. Analyses were restricted to 352 unique patients.

## 4 Discussion

This study provides one of the longest longitudinal evaluations of antimicrobial resistance in *P. aeruginosa* from a Saudi secondary healthcare setting, describing seven-year trends in clinically important resistance phenotypes. Overall, MDR and XDR prevalence differed significantly across the study period, whereas DTR did not demonstrate significant temporal variation. Importantly, the associations between collection year and both MDR and XDR remained significant after adjustment for age group, sex, nationality, and specimen source, suggesting that the observed temporal differences were not explained solely by changes in these measured case-mix characteristics. These findings reinforce the substantial burden of antimicrobial resistance in *P. aeruginosa* and are broadly consistent with recent national surveillance data from Saudi Arabia, although direct comparisons should be interpreted cautiously because of differences in study design, isolate selection, susceptibility testing methods, and resistance definitions (Thabit et al., 2024).

The temporal pattern observed in this study was characterized by substantial year-to-year variation in MDR and XDR prevalence, with both phenotypes reaching their highest levels during the later study years before declining in 2024. These fluctuations may reflect the combined influence of antimicrobial prescribing practices, infection prevention measures, and evolving healthcare utilization. Although an antimicrobial stewardship program was introduced at the study institution during the surveillance period, the observational design precludes attributing the observed changes to any specific intervention. Nevertheless, these findings underscore the importance of continuous longitudinal surveillance to monitor evolving resistance patterns and evaluate stewardship efforts over time.

Respiratory tract specimens were the most common source of isolates in our cohort. This pattern is consistent with the established role of *P. aeruginosa* as a major pathogen in healthcare-associated respiratory infections, including ventilator-associated pneumonia and lower respiratory tract infection in hospitalized patients. Large surveillance studies have likewise reported respiratory specimens among the most frequent sources of *P. aeruginosa* isolates in hospital settings (Elfadadny et al., 2024). The predominance of older adults in our study population is also clinically plausible, as advanced age is associated with greater healthcare exposure, comorbidity burden, and increased risk of *P. aeruginosa* infection in respiratory disease settings (Elfadadny et al., 2024; Wijit et al., 2023).

Beyond the observed resistance phenotypes, detailed antimicrobial non-susceptibility data (**Supplementary Table S1**) demonstrated substantial resistance across several clinically important antipseudomonal agents throughout the study period. Although individual resistance patterns varied by antimicrobial class and year, the high prevalence of MDR and XDR isolates across several study years indicates a substantial burden of resistance that may restrict therapeutic options. Similar resistance profiles have been reported in recent Saudi and regional surveillance studies, where resistance to carbapenems, cephalosporins, and fluoroquinolones remains a major therapeutic challenge (Alatoom et al., 2024; Kadri et al., 2018; Thabit et al., 2024; El-Far and Abukhatwah, 2023).

The substantial year-to-year variation in MDR and XDR prevalence observed in this study highlights the dynamic nature of antimicrobial resistance in *P. aeruginosa*. In contrast, DTR prevalence fluctuated over the study period without statistically significant differences across years. Among these phenotypes, DTR remains particularly important because it reflects loss of susceptibility to the principal first-line antipseudomonal β-lactams and fluoroquinolones, thereby identifying isolates with substantially restricted treatment options. The DTR framework proposed by Kadri et al. was developed to better capture this clinically relevant therapeutic challenge and has been associated with adverse outcomes in patients with Gram-negative infections (Kadri et al., 2018).

This study has several strengths. It provides seven years of longitudinal resistance surveillance from a secondary healthcare setting, applies a deduplicated isolate approach to reduce bias from repeated sampling, and includes the DTR classification in addition to MDR and XDR, thereby offering a clinically relevant perspective on resistance surveillance. The incorporation of temporal trend analysis using logistic regression further strengthened the evaluation by quantifying changes in resistance phenotypes over time. Several limitations should also be considered. The study was retrospective and laboratory-based, and detailed clinical data such as antimicrobial exposure, comorbidities, treatment received, and patient outcomes were not available; therefore, infection could not be reliably distinguished from colonization for all isolates. In addition, the study was conducted at a single center and molecular characterization of isolates was not performed; therefore, the observed phenotypic trends could not be attributed to clonal expansion or the acquisition and dissemination of specific resistance determinants. Annual isolate counts varied across the study period, and the available data did not allow us to determine whether this reflected changes in patient volume, clinical sampling practices, or other healthcare-related factors. The relatively small and variable annual denominators should therefore be considered when interpreting year-to-year fluctuations in resistance prevalence. An additional consideration is that susceptibility was interpreted using the CLSI breakpoints in effect at the time of testing; therefore, breakpoint revisions during the study period may have introduced some variability in longitudinal resistance classification. Although MDR, XDR, and DTR definitions were applied consistently throughout the study, retrospective application of a single fixed breakpoint set was not feasible with the susceptibility data available for analysis. Although overall susceptibility-data completeness was high (92.5%), incomplete testing for some isolates may also have affected resistance phenotype classification. Furthermore, although the temporal analyses accounted for available demographic and specimen characteristics, residual confounding by unmeasured clinical factors remains possible.

In conclusion, this single-center study demonstrates a substantial burden of antimicrobial resistance among P. aeruginosa isolates in a Saudi secondary healthcare setting. The significant year-to-year variation in MDR and XDR prevalence highlights the dynamic nature of phenotypic antimicrobial resistance during the study period. These findings provide locally relevant surveillance data that may inform empirical antimicrobial therapy, antimicrobial stewardship programs, and ongoing institutional surveillance.

## Data Availability

The datasets presented in this article are not readily available due to institutional and ethical restrictions. Requests to access the datasets should be directed to walghamdi@kku.edu.sa.

## References

[B1] Alatoom A. Alattas M. Alraddadi B. Ayoub Moubareck C. Hassanien A. Jamal W. . (2024). Antimicrobial resistance profiles of *Pseudomonas aeruginosa* in the Arabian Gulf region over a 12-year period, (2010-2021). J. Epidemiol. Global Health 14, 529–548. doi: 10.1007/s44197-024-00191-y 38856819 PMC11442796

[B2] Antimicrobial Resistance Collaborators (2022). Global burden of bacterial antimicrobial resistance in 2019: A systematic analysis. Lancet (London England) 399, 629–655. doi: 10.1016/S0140-6736(21)02724-0 35065702 PMC8841637

[B3] Clinical and Laboratory Standards Institute (2025). “ CLSI supplement M100,” in Performance Standards for Antimicrobial Susceptibility Testing, 35th ed ( Clinical and Laboratory Standards Institute, Wayne, PA).

[B4] Elfadadny A. Ragab R. F. AlHarbi M. Badshah F. Ibáñez-Arancibia E. Farag A. . (2024). Antimicrobial resistance of *Pseudomonas aeruginosa*: Navigating clinical impacts, current resistance trends, and innovations in breaking therapies. Front. Microbiol. 15, 1374466. doi: 10.3389/fmicb.2024.1374466 38646632 PMC11026690

[B5] El-Far S. W. Abukhatwah M. W. (2023). Prevalence of aminoglycoside resistance genes in clinical isolates of *Pseudomonas aeruginosa* from Taif, Saudi Arabia-an emergence indicative study. Microorganisms 11, 2293. doi: 10.3390/microorganisms11092293 37764137 PMC10537265

[B6] Kadri S. S. Adjemian J. Lai Y. L. Spaulding A. B. Ricotta E. Prevots D. R. . (2018). Difficult-to-treat resistance in gram-negative bacteremia at 173 US hospitals: Retrospective cohort analysis of prevalence, predictors, and outcome of resistance to all first-line agents. Clin. Infect. Diseases: An. Off. Publ. Infect. Dis. Soc. America 67, 1803–1814. doi: 10.1093/cid/ciy378 30052813 PMC6260171

[B7] Magiorakos A. P. Srinivasan A. Carey R. B. Carmeli Y. Falagas M. E. Giske C. G. . (2012). Multidrug-resistant, extensively drug-resistant and pandrug-resistant bacteria: An international expert proposal for interim standard definitions for acquired resistance. Clin. Microbiol. Infection: Off. Publ. Eur. Soc. Clin. Microbiol. Infect. Dis. 18, 268–281. doi: 10.1111/j.1469-0691.2011.03570.x 21793988

[B8] Mancuso G. Midiri A. Gerace E. Biondo C. (2021). Bacterial antibiotic resistance: The most critical pathogens. Pathog. (Basel Switzerland) 10, 1310. doi: 10.3390/pathogens10101310 34684258 PMC8541462

[B9] Moradali M. F. Ghods S. Rehm B. H. A. (2017). *Pseudomonas aeruginosa* lifestyle: A paradigm for adaptation, survival, and persistence. Front. Cell. Infect. Microbiol. 7, 39. doi: 10.3389/fcimb.2017.00039 28261568 PMC5310132

[B10] Saudi Public Health Authority (2022). Kingdom of Saudi Arabia National Action Plan for Antimicrobial Resistance 2022–2025 (Riyadh: Saudi Public Health Authority). Available online at: https://cdn.who.int/media/docs/default-source/antimicrobial-resistance/amr-spc-npm/nap-library/kingdom-of-saudi-arabia-nap-amr-2022-2025.pdf (Accessed July 26, 2026).

[B11] Thabit A. K. Alghamdi A. M. Miaji M. Y. Alharbi F. S. Jawah A. F. Alturki F. . (2024). Antibiotic susceptibility of *Pseudomonas aeruginosa* in Saudi Arabia: A national antimicrobial resistance surveillance study. Front. Public Health 12, 1436648. doi: 10.3389/fpubh.2024.1436648 39403434 PMC11471531

[B12] Wijit K. Sonthisombat P. Diewsurin J. (2023). A score to predict *Pseudomonas aeruginosa* infection in older patients with community-acquired pneumonia. BMC Infect. Dis. 23, 700. doi: 10.1186/s12879-023-08688-w 37858082 PMC10585923

