# Supplementary material for: Longitudinal trends in antimicrobial resistance phenotypes in Pseudomonas aeruginosa: a seven-year study
Source: Front Antibiot. 2026 Sep 18;5:1912287. doi: 10.3389/frabi.2026.1912287 (PMC13630743; doi:10.3389/frabi.2026.1912287)
Supplement: Supplementary file 1 [file Table1.docx]

Supplementary Material

**Table S1.** Annual antimicrobial non-susceptibility of P. aeruginosa isolates by antimicrobial agent, 2018–2024.

| **Antimicrobial agent** | **2018** | **2019** | **2020** | **2021** | **2022** | **2023** | **2024** |
| --- | --- | --- | --- | --- | --- | --- | --- |
| Piperacillin/ tazobactam | 22 (44.9) | 18 (40.9) | 24 (49.0) | 35 (51.5) | 23 (47.9) | 25 (65.8) | 16 (34.8) |
| Imipenem | 21 (45.7) | 17 (35.4) | 31 (59.6) | 44 (59.5) | 27 (50.0) | 23 (56.1) | 14 (30.4) |
| Meropenem | 21 (41.2) | 15 (34.1) | 26 (52.0) | 41 (56.2) | 28 (51.9) | 18 (46.2) | 12 (27.3) |
| Cefepime | 25 (50.0) | 18 (38.3) | 24 (46.2) | 41 (56.9) | 34 (63.0) | 25 (61.0) | 19 (43.2) |
| Ceftazidime | 23 (52.3) | 18 (40.0) | 26 (52.0) | 41 (55.4) | 31 (57.4) | 25 (61.0) | 18 (40.9) |
| Aztreonam | 24 (57.1) | 22 (47.8) | 32 (61.5) | 50 (69.4) | 36 (70.6) | 26 (66.7) | 28 (62.2) |
| Ciprofloxacin | 22 (44.0) | 12 (28.6) | 24 (47.1) | 38 (50.7) | 47 (85.5) | 41 (100) | 45 (97.8) |
| Levofloxacin | 17 (35.4) | 8 (20.5) | 24 (48.0) | 35 (51.5) | 41 (75.9) | 40 (100) | 45 (97.8) |
| Amikacin | 13 (25.5) | 11 (22.9) | 23 (44.2) | 36 (48.6) | 27 (49.1) | 18 (46.2) | 9 (20.0) |
| Gentamicin | 16 (31.4) | 18 (37.5) | 30 (57.7) | 46 (62.2) | 32 (58.2) | 24 (58.5) | 15 (32.6) |
| Tobramycin | 16 (31.4) | 12 (26.1) | 25 (48.1) | 36 (49.3) | 28 (52.8) | 16 (40.0) | 10 (24.4) |
| Colistin | 9 (23.1) | 6 (14.3) | 15 (30.0) | 21 (34.4) | 39 (76.5) | 31 (100) | 33 (100) |

Data are presented as n (%). Non-susceptibility includes isolates categorized as intermediate or resistant.
